# Cell Wall Invertases from Maternal Tissues Modulate Sucrose Flux in Apoplastic Pathways During Rice Anther and Seed Development

**DOI:** 10.3390/ijms252111557

**Published:** 2024-10-28

**Authors:** Sang-Kyu Lee, Su-Hyeon Shim, Joon-Seob Eom, Jung-Il Cho, Jae-Ung Kwak, Seong-Cheol Eom, Jong-Seong Jeon

**Affiliations:** 1 Division of Life Science, Plant Molecular Biology and Biotechnology Research Center, Gyeongsang National University, Jinju 52828, Republic of Korea; jw0707911@daum.net (J.-U.K.); esc2455@gmail.com (S.-C.E.); 2 Graduate School of Green-Bio Science and Crop Biotech Institute, Kyung Hee University, Yongin 17104, Republic of Korea; hiyaho15@khu.ac.kr (S.-H.S.); june031100@gmail.com (J.-S.E.); jungilcho@korea.kr (J.-I.C.); 3 Crop Production and Physiology Division, National Institute of Crop Science, Rural Development Administration, Wanju 55365, Republic of Korea

**Keywords:** cell wall invertase, male sterility, shrunken seed, *Oryza sativa*, anther, phloem unloading

## Abstract

Efficient sucrose transport and metabolism are vital for seed and pollen development in plants. Cell wall invertases (CINs) hydrolyze sucrose into glucose and fructose, maintaining a sucrose gradient in the apoplast of sink tissues. In rice, two CIN isoforms, OsCIN1 and OsCIN2, were identified as being specifically expressed in the anthers but not in pollen. Functional analyses through genetic crosses and mutant characterization showed that *oscin1/2* double mutants exhibit a sporophytic male-sterile phenotype and produce shrunken seeds. This suggests that CIN activity is essential for proper pollen development and seed formation in rice. Observation of the progeny genotypes and phenotypes from various genetic crosses revealed that the phenotype of *oscin1/2* seeds is determined by the genotype of the maternal tissue, indicating the critical role of CIN function in the apoplast between maternal and filial tissues for sucrose transport and metabolism. The CIN activity in the anthers and seeds of wild-type rice was found to be significantly higher—over 500-fold in the anthers and 5-fold in the seeds—than in the leaves, highlighting the importance of CIN in facilitating the efficient unloading of sucrose. These findings suggest that the fine-tuning of CIN activity in the apoplast, achieved through tissue-specific expression and CIN isoform regulation, plays a key role in determining the carbohydrate distribution across different tissues. Understanding this regulatory mechanism could provide opportunities to manipulate carbohydrate allocation to sink organs, potentially enhancing crop yields.

## 1. Introduction

The transport of sugars from source to sink tissues is critical for plant development. Carbohydrates synthesized in photosynthetically active source tissues are transported to sink tissues as sucrose via the phloem. Plants utilize two main enzymes for sucrose cleavage: sucrose synthase, which catalyzes a reversible reaction cleaving sucrose into UDP-glucose and fructose, and invertase, which irreversibly hydrolyzes sucrose into glucose and fructose [1]. In higher plants, invertases are classified based on their subcellular localization into cytoplasmic, vacuolar, and cell wall invertases (CINs) [2]. Among these, CINs are the only ones localized in the apoplast, making them key enzymes in sucrose unloading and sink tissue development [3].

Sucrose is synthesized in mesophyll cells and subsequently transported toward the phloem. SWEET (Sugars Will Eventually be Exported Transporter) proteins, a family of pH-independent uniporters, facilitate the transport of sugars, such as glucose and sucrose, across cellular membranes. These transporters play a crucial role in exporting sucrose from the symplast into the apoplast, enabling its uptake by sucrose transporters (SUTs) into the sieve element–companion cell complex [4]. In Arabidopsis, double mutants for AtSWEET11 and AtSWEET12, which are highly expressed in leaves, showed reduced sucrose export and starch accumulation, highlighting the essential role of these proteins in phloem loading [5]. In rice, double mutants for OsSWEET11a and OsSWEET11b showed male sterility and a shrunken-seed phenotype [6]. The expression of OsSWEET11a and OsSWEET11b in the veins of the anthers further supports their role in unloading sucrose into the apoplast.

Because the pollen grains and filial tissues within seeds are symplastically isolated from surrounding maternal tissues, the carbohydrates required for their development must be imported through the apoplast. Several studies have confirmed the expression and function of CINs, which are expected to act in the apoplasts of sink tissues. In tobacco, the antisense repression of a CIN expressed in the pollen led to male sterility [7,8]. Further studies demonstrated that CIN expressed in maternal tissues plays a major role in sustaining the energy-intensive growth of the pollen tube [9]. In wheat, under water-deficit conditions, reduced expression of an anther-specific CIN was observed, indicating its critical role in anther function and fertility [10]. Similarly, in tomato, decreased expression of the CIN isoform Lin7, which is expressed in pollen and pollen tubes, resulted in impaired pollen development, underscoring the necessity of CINs in reproductive growth [11]. In oilseed rape, the antisense repression of a CIN also led to defects in pollen development [12]. The mutation of the apoplastic invertase gene, INCW2, in maize resulted in a miniature seed phenotype, highlighting the importance of this enzyme in seed development [13,14]. Similarly, the rice CIN mutant *gif1* (grain incomplete filling 1) exhibited an opaque seed phenotype, further confirming the essential role of invertases in seed development [15].

To date, no functional studies on CINs in rice have been conducted, except for *gif1*. In this study, we identified two CIN isoforms, *OsCIN1* and *OsCIN2*, which are specifically expressed in the anthers but not in pollen during pollen development. Through the phenotypic analysis of *oscin1/2* double mutants and comparisons of genotypes and phenotypes from various genetic crosses, we demonstrated the critical role of CIN activity in sucrose transport and metabolism. Our findings reveal that CINs function within the apoplast between maternal and filial tissues, playing an essential role in both pollen development and seed formation. This study underscores the importance of CIN regulation in maintaining proper carbohydrate distribution to reproductive tissues, which has significant implications for seed fertility and crop yield.

## 2. Results

### 2.1. Identification of Rice CIN Genes Preferentially Expressed in Anthers and Pollen Grains

To ensure a continuous supply of sugar to rice pollen grains, it is essential that sucrose is consistently hydrolyzed in the apoplast between the maternal tissues and pollen grains, maintaining a sucrose gradient between them to facilitate efficient sugar transport into the pollen. Among the potential pathways, sucrose degradation in the apoplastic space by CIN is a highly plausible mechanism by which this gradient might be sustained. Through analysis of the rice genome, nine *OsCIN* genes were identified. We examined the expression patterns of these genes using digital Northern analysis. Among them, LOC_Os02g33110 (*OsCIN1*) and LOC_Os04g33740 (*OsCIN2*) were expressed exclusively in the anthers, while LOC_Os04g33720 (*OsCIN3*) and LOC_Os09g08072 (*OsCIN7*) were expressed in both the anthers and pollen grains (Figure S1).

**Figure 1:**
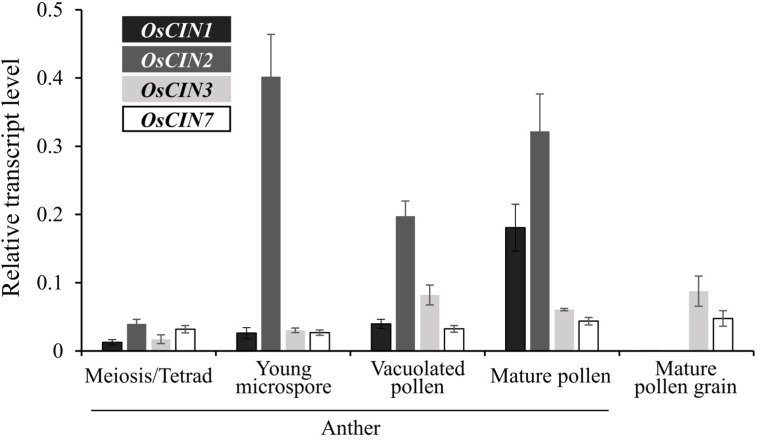
Quantitative RT-PCR analysis of cell wall invertase (*OsCIN*) gene expression in anthers from the meiosis stage to mature pollen, as well as in mature pollen grains. Data represent the means (±SEs) of three replicates. Data were normalized using the *OsUBQ5* gene as an internal control, and the results are expressed as relative values.

RT-qPCR analysis further confirmed the expression patterns of *OsCIN* genes in the anthers during pollen development, which showed results highly consistent with those of the digital Northern analysis (Figure 1). Due to the difficulty of isolating pollen from the anther tissues at earlier developmental stages, we conducted the analysis using entire anthers. *OsCIN1* exhibited the highest expression in the mature pollen stage, while *OsCIN2* showed strong expression starting from the young microspore stage, continuing up to the mature pollen stage. Notably, neither *OsCIN1* nor *OsCIN2* was expressed in mature pollen grains, which can be isolated from the anthers at the mature stage. Additionally, although *OsCIN3* and *OsCIN7* were detected in the anthers during pollen development, their expression was very low. *OsCIN3* showed a slight increase in expression in the anthers during the vacuolated pollen stage. It was confirmed that the accumulation of both *OsCIN3* and *OsCIN7* transcripts originated from the pollen grains rather than the anthers (Figure 1).

### 2.2. oscin1/2 Double Mutant Exhibits Male Sterility Phenotype

**Figure 2:**
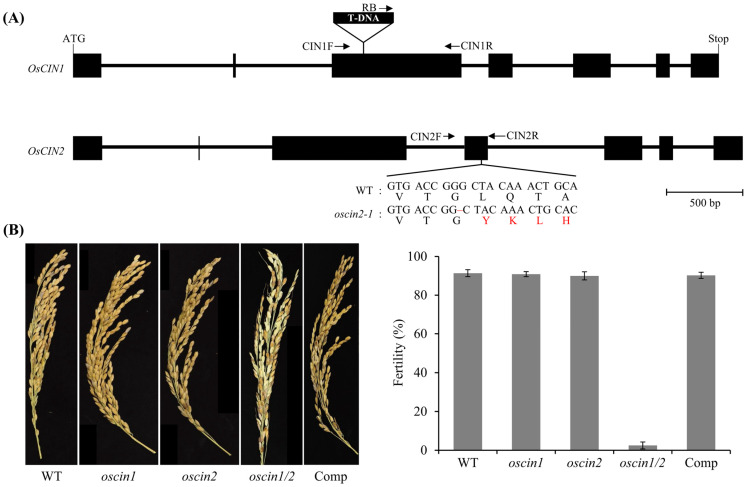
Phenotypes of *OsCIN* mutants. (**A**) Schematic diagrams of the *OsCIN1* and *OsCIN2* genes and their mutations. In *oscin1*, T-DNA is inserted into the third exon of *OsCIN1*. The *gif1* mutant, with a single-nucleotide deletion (shown in red), causes premature termination and was used as the *oscin2* allele. The exons of each gene are indicated by filled boxes, and the primers for genotyping are marked with arrows. WT represents the mutant background genotype of cv. Dongjin for *oscin1.* (**B**) Fertility of *OsCIN* mutants. Phenotypic analysis of fully dried panicles (**left**) and the corresponding quantitative fertility rates (**right**). In fully dried panicles, a few brown and matured spikelets bore seeds in *oscin1*/*2*. Each value represents the mean (±SE) of 10 panicles from at least five plants.

To analyze the function of the two *OsCIN* genes preferentially expressed in the anthers, *OsCIN1* and *OsCIN2*, we isolated mutants for both genes. For *OsCIN1*, we isolated a complete loss-of-function mutant line, *oscin1*, which harbors a T-DNA insertion in the third exon (Figure 2A). In addition, the previously published *gif1* mutant, described as a null mutant of *OsCIN2* due to a single-nucleotide deletion in the fourth exon causing a frameshift, was designated as *oscin2* [15]. Neither of the single mutants exhibited any visible phenotypes in their anthers or pollen grains. Based on our gene expression analysis (Figure 1 and Figure S1), we hypothesized that the lack of observable phenotypes in the single mutants could have been due to functional redundancy between *OsCIN1* and *OsCIN2*. Both genes are strongly expressed in the anther tissues at the mature pollen stage, suggesting they may compensate for each other’s functions. Given that neither single mutant displayed significant phenotypic changes, we suspected that these two genes might perform similar roles in sucrose metabolism during pollen development. To test this hypothesis, we generated an *oscin1/2* double mutant through genetic crossing. Remarkably, *oscin1/2* displayed a significant reduction in seed set, with the rates dropping to approximately 2% (Figure 2B), supporting the idea that *OsCIN1* and *OsCIN2* have overlapping functions in the anthers.

To determine whether the double mutation of *OsCIN1* and *OsCIN2* was responsible for the male sterility phenotype, we introduced *OsCIN1* cDNA under the control of the CaMV 35S promoter (*p35S*) into japonica rice cv. Dongjin via *Agrobacterium*-mediated transformation. After selecting three transgenic plants overexpressing *OsCIN1*, they were crossed with *oscin1/2* double mutants. Progeny from the F1 plants were obtained, and those with the *oscin1/oscin2/p35S:OsCIN1* genotype were identified using genomic DNA PCR for *OsCIN1* and sequencing for *OsCIN2*. Finally, *OsCIN1* cDNA-carrying *oscin1/2* double-homozygous plants were selected and designated as Comp lines. The genotypes of and *OsCIN1* expression levels in the Comp plants were confirmed (Figure S2). Among the lines, Comp #2, which had the highest expression of the introduced *OsCIN1* transgene, was chosen for further analysis. The seed-setting rate of the Comp #2 line was comparable to the setting rates of WT, *oscin1*, and *oscin2* (Figure 2B), suggesting that the overexpression of *OsCIN1* can rescue seed fertility in *oscin1/2*.

### 2.3. Pollen Phenotype of oscin1/2 Double Mutant

**Figure 3:**
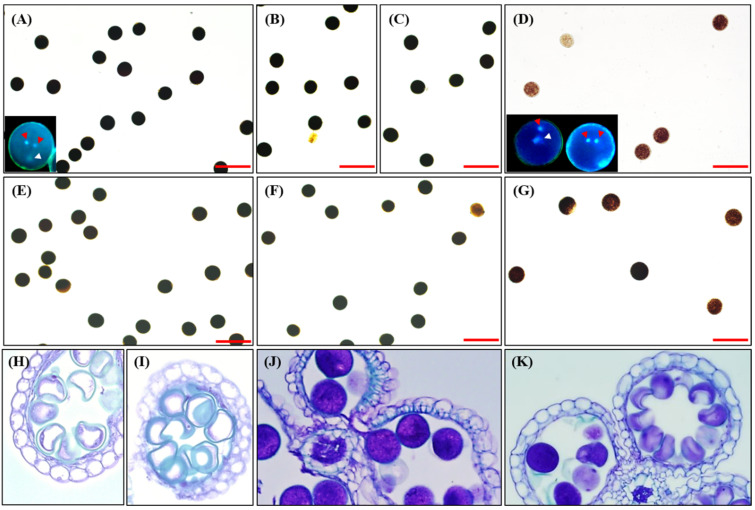
Pollen phenotypes of *oscin1*/*2* mutants. (**A**–**G**) Iodine-stained pollen grains of *oscin1*/*2* mutants grown under 14 h light conditions. (**A**) WT. (**B**) *oscin1.* (**C**) *oscin2.* (**D**) *oscin1/2.* (**E**) *oscin1/OsCIN2^+/−^.* (**F**) *OsCIN1^+/−^/oscin2.* (**G**) Complemented line, *oscin1/oscin2/p35S:OsCIN1.* Scale bars = 50 μm. (**H**–**K**) Cross-sections of *oscin1*/*2* anthers at the meiosis stage for (**H**) WT and (**I**) *oscin1*/*2* and at the mature pollen stage for (**J**) WT and (**K**) *oscin1*/*2*. Pollen grains stained with Hoechst 33342 are shown in the insets of (**A**,**D**), where red arrowheads indicate generative nuclei and white arrowheads indicate vegetative nuclei.

Given the low seed-setting rates observed in *oscin1/2*, we hypothesized that abnormalities in pollen development could contribute to this phenotype. To investigate this, we performed iodine staining to assess pollen viability. Mature pollen grains from WT, *oscin1*, and *oscin2* all showed normal staining (Figure 3A–C). However, in *oscin1/2*, a considerable number of pollen grains exhibited weak staining (Figure 3D). Typically, in heterozygous plants that are gametophytic male-sterile mutants, half of the pollen is expected to be normal, while the other half displays the mutant phenotype. In homozygous plants, all pollen should exhibit the mutant phenotype. Notably, in *oscin1/OsCIN2^+/−^* and *OsCIN1^+/−^/oscin2* plants, none of the pollen grains displayed any defective phenotypes (Figure 3E,F). These results suggest that *oscin1/2* is not a typical gametophytic mutant, where the pollen phenotype is determined by the pollen’s genotype, but rather a mutant in which the male sterility phenotype is determined by the genotype of the maternal tissue. Further morphological analysis of the pollen from the WT and *oscin1/2* at the unicellular microspore stage (stages 9 and 10) and the mature pollen stage (stage 13), as described by Lee et al. [16], revealed that pollen development in *oscin1/2* was similar to that in the WT at the unicellular microspore stage (Figure 3H,I). However, some pollen grains from *oscin1/2* appeared to be arrested at the bicellular or tricellular stage (stages 11 and 12), failing to develop into mature pollen (Figure 3J,K). Nuclear staining confirmed that the *oscin1/2* plants contained a mixture of pollen grains at the bicellular and tricellular stages (Figure 3A,D). All the results, including those from starch content analysis, morphological assessments, and nuclear staining of *oscin1/2* pollen grains, consistently indicate irregularities in pollen development. When the pollen grains of Comp #2 were stained with iodine, a greater number of stained pollen grains were observed compared to the quantity for the *oscin1/2* double mutant (Figure 3G). However, unlike for the WT, not all the pollen grains were fully stained. Given the rescued seed-setting rate of the Comp #2 line (Figure 2B), these results suggest that the overexpression of *OsCIN1* can partially rescue delayed pollen development phenotype in *oscin1/2*, allowing a sufficient number of pollen grains to fully mature and accumulate starch (Figure 3G). Taken together, these findings clearly demonstrate that the phenotype of *oscin1/2* pollen is not determined by the genotype of individual pollen grains, as would be expected in a typical gametophytic mutant. Instead, the observed defects in pollen development and the male sterility phenotype are determined by the genotype of the maternal tissue, which plays a key role in regulating the sucrose metabolism necessary for proper pollen maturation and starch accumulation. This suggests that disrupted sucrose transport in the maternal tissues of *oscin1/2* mutants impairs pollen development, resulting in the delayed or incomplete maturation of pollen grains.

### 2.4. Seed Phenotype of oscin1/2 Double Mutant

**Figure 4:**
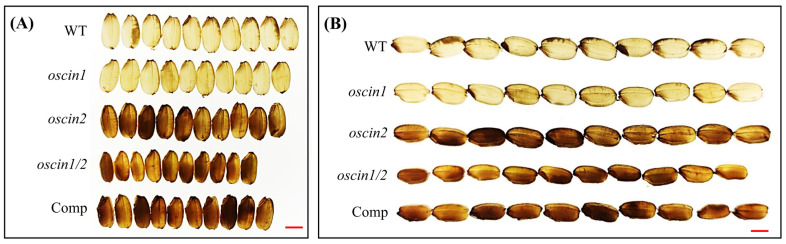
Phenotypes of seeds of *OsCIN* mutants observed on a lightbox. Opaque seeds exhibit a non-transparent appearance. (**A**) Widths of seeds of *OsCIN* mutants. (**B**) Lengths of seeds of *OsCIN* mutants. Scale bars = 2 mm.

A comparison of the seed sizes among *oscin1*, *oscin2*, *oscin1/2*, Comp #2, and WT revealed that the *oscin1/2* mutant seeds were the smallest. The *oscin1/2* seeds exhibited a significant reduction in width and a slight decrease in length, as well as a reduction in seed weight, with some seeds displaying a severely shrunken phenotype (Figure 4A,B and Figure S3). The seeds of *oscin1* showed no differences compared to those of the WT, while the previously reported opaque seed phenotype was observed for *oscin2* [15]. Similarly, *oscin1/2* seeds also exhibited the opaque seed phenotype. In Comp #2, the seed size was partially restored, but the opaque seed phenotype persisted (Figure 4A,B). This suggests that while the overexpression of *OsCIN1* can complement the function of *OsCIN1* in *oscin1/2* seeds, it fails to restore *OsCIN2* function, likely due to the tissue-specific expression of *OsCIN2* despite the similar enzymatic functions of the two CINs.

During the generation of *oscin1/2* through genetic crossing, an interesting phenomenon was observed. Among the self-fertilized seeds of *OsCIN1^+/–^/oscin2* or *oscin1/OsCIN2^+/−^* plants, none exhibited the shrunken-seed phenotype of *oscin1/2*, even though 25% of them should theoretically have done so. Additionally, the segregation ratio of the genotypes in the self-fertilized seeds of *OsCIN1^+/−^/oscin2* or *oscin1/OsCIN2^+/−^* plants followed the expected Mendelian ratio of 1:2:1. This segregation would not be possible if male gametes with the *oscin1/2* genotype were unable to participate normally in fertilization. However, when *oscin1/2* double mutant plants were self-fertilized, all the resulting seeds exhibited the shrunken-seed phenotype. Moreover, due to the very low fertility of *oscin1/2*, cross-contamination with surrounding plants was common. Even when the genotype of these cross-contaminated seeds was *OsCIN1^+/−^/OsCIN2^+/−^*, they still exhibited the shrunken-seed phenotype of the *oscin1/2* double mutant.

To further investigate this, we analyzed the genotypes and phenotypes of various progenies from different crosses (Table 1). As predicted, when self-pollinating a heterozygous *oscin1/OsCIN2^+/−^* plant, the expected segregation ratio of 25% *oscin1/OsCIN2^+/+^*, 50% *oscin1/OsCIN2^+/−^*, and 25% *oscin1/oscin2* was observed. However, despite their genotypes, all the seeds exhibited a normal phenotype similar to *oscin1* seeds. When self-pollinating an *OsCIN1^+/−^/oscin2* plant, the expected segregation of 25% *OsCIN1^+/+^/oscin2*, 50% *OsCIN1^+/−^/oscin2*, and 25% *oscin1/oscin2* was observed, but all displayed an opaque seed phenotype similar to *oscin2* seeds, even though the *oscin1/oscin2* genotype could theoretically result in a shrunken-seed phenotype. Similarly, when self-pollinating an *oscin1/oscin2/p35S:OsCIN1^+/−^* plant, the expected segregation of 75% *oscin1/oscin2/p35S:OsCIN1^+/+^* or *oscin1/oscin2/p35S:OsCIN1^+/−^* and 25% *oscin1/oscin2/p35S:OsCIN1^−/−^* was observed. Even the seeds with the *oscin1/oscin2/p35S:OsCIN1^−^* genotype, which were expected to exhibit a shrunken-seed phenotype, displayed no morphological differences, except for an opaque seed phenotype similar to that for the *oscin2* seeds.

This discrepancy between seed genotype and phenotype strongly suggests that the shrunken-seed phenotype of *oscin1/2* is determined not by the genotype of the newly fertilized seeds but by the genotype of the maternal plant. The seed phenotypes observed from various crosses strongly support this hypothesis. To further confirm this, we conducted a cross using the WT as the pollen donor and *oscin1/2* as the maternal parent. As expected, despite all the F1 seeds having the genotype *OsCIN1^+/−^/OsCIN2^+/−^*, they exhibited the shrunken phenotype (Table 1).

### 2.5. CIN Activity in Anthers and Seeds of OsCIN Mutants

**Figure 5:**
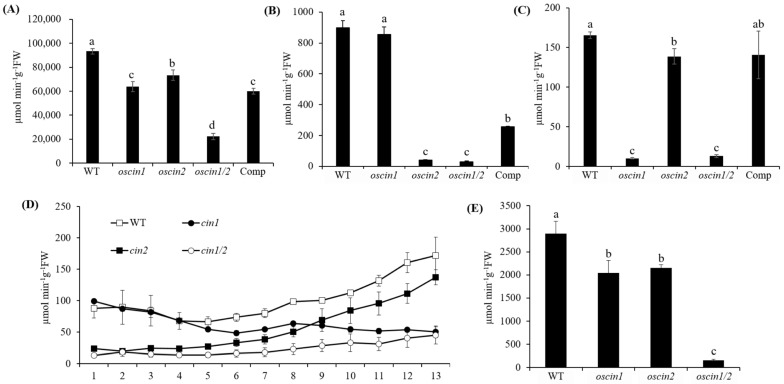
Measurement of cell wall invertase activity in different tissues. CIN activity in (**A**) anthers at the mature pollen stage, (**B**) seeds at 3 days after fertilization, (**C**) the third leaf from the top in 6-week-old plants, (**D**) stems of 6-week-old plants, and (**E**) primary and secondary panicle branches. In (**D**), the x-axis numbers represent the lengths from the base of the stem: 1 indicates 0–1 cm from the base, 2 indicates 1–2 cm, etc., with 13 indicating 12–13 cm from the base. All the data represent means (±SEs) from at least three different plants. Significant differences between groups were determined using Tukey’s HSD (Honestly Significant Difference) test for multiple comparisons. Different lowercase letters indicate significant differences (*p* < 0.05).

The CIN activity was measured in each *OsCIN* mutant, including *oscin1*, *oscin2*, *oscin1/2*, and Comp #2. The CIN activity in the anthers of both the *oscin1* and *oscin2* mutants was significantly lower than that in the WT, with the activity in the *oscin1/2* double mutant being further reduced compared to that in each single mutant. These results suggest that both OsCIN1 and OsCIN2 contribute to the overall CIN activity in the anthers. In the complement line, Comp #2, CIN activity was observed at a level similar to that of *oscin1* (Figure 5A). This finding aligns with the reduction in the number of stained pollen grains and the decrease in seed fertility, which was most pronounced in *oscin1/2* (Figure 2B). *oscin1* did not show a significant reduction in CIN activity compared to the WT. However, *oscin2* exhibited very low CIN activity, and similarly, the activity remained very low in *oscin1/2*, indicating that OsCIN2 is crucial for CIN activity in seeds. In Comp #2, the CIN activity was higher than that in *oscin1/2* but did not reach WT levels (Figure 5B). This observation is consistent with the opaque seed phenotype observed in *oscin2* (Figure 4B).

Notably, the CIN activity in the WT seeds and anthers was approximately 5 times and over 500 times higher, respectively, than that in the WT leaves. This supports the hypothesis that various *OsCINs* function to rapidly break down sucrose transported from the leaves to sink organs, thereby lowering the sucrose concentrations in sink tissues. In *oscin1*, the CIN activity in the leaves was greatly reduced, while in *oscin2*, a slight but significant reduction was observed compared to the WT (Figure 5C). This indicates that *OsCIN1* plays a major role in the leaves. However, CIN activity in the leaves does not seem to significantly affect plant growth, seed size, or fertility.

In the stem, we observed that the CIN activity in the WT progressively increased from the lower to upper parts (Figure 5D). The upper part of the stem did not photosynthesize but continued to receive carbohydrates for rapid growth, likely explaining the high CIN activity in this region. In *oscin1/2*, the CIN activity was significantly reduced in all parts of the stem. In contrast, in *oscin2*, the CIN activity increased toward the upper part of the stem, similar to the WT, but at a lower level (Figure 5D). This suggests that OsCIN2 activity is consistently present throughout the stem, while OsCIN1 activity increases toward the upper part. In the panicle branch, a pattern of activity similar to that in the anthers was observed, but at lower levels. In the panicle branches of both *oscin1* and *oscin2*, the CIN activity was lower than that in the WT, and in *oscin1/2*, the activity was significantly lower than that in the WT and in each single mutant. Overall, the CIN activity in WT panicle branches was approximately 30 times lower than that in the anthers but more than 17 times higher than that in the leaves (Figure 5E).

Overall, these findings highlight the gradient of CIN activity across various tissues, with lower activity in the leaves, increasing progressively from the lower to upper part of the stem, and peaking in the anthers, followed by significant activity in seeds. This variation in CIN activity suggests a crucial role in facilitating the transport of sucrose from the source tissues, such as leaves, to the sink organs, including the anthers and seeds. The coordinated activity of OsCIN1 and OsCIN2 ensures efficient sucrose unloading and metabolism, which is essential for proper pollen and seed development, as well as overall plant growth.

### 2.6. Carbohydrate Flow in Reproductive Organs of OsCIN Mutants

**Figure 6:**
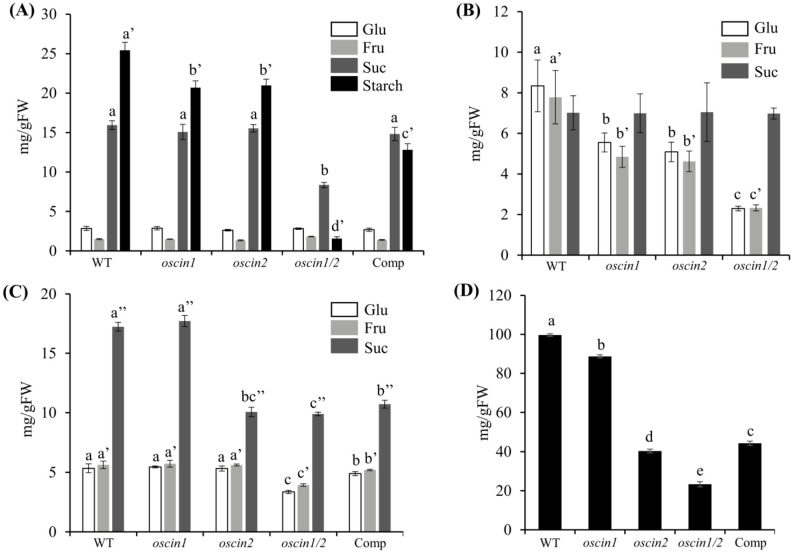
Determination of soluble sugar and starch. (**A**) Soluble sugar and starch contents in mature anthers of WT and *OsCIN* mutants. (**B**) Soluble sugar content in the locular fluid of WT and *OsCIN* mutants. (**C**) Soluble sugar content in seeds at 3 days after fertilization from WT and *OsCIN* mutants. (**D**) Starch content in seeds at 3 days after fertilization from WT and *OsCIN* mutants. All the data represent means (±SEs) from at least three different plants. Significant differences between groups were determined using Tukey’s HSD (Honestly Significant Difference) test for multiple comparisons. Different lowercase letters indicate significant differences (*p* < 0.05). The number of apostrophes following each letter represents the groups compared in the statistical analysis.

Following the observation of reduced starch levels in the *oscin1/2* mutant via iodine staining, we quantified the starch and soluble sugar levels in the anthers. Ideally, pollen grains should be isolated and measured separately for accurate assessment, but due to the difficulty of isolating pollen, we conducted the analysis using entire anthers. Consistent with the staining results, we observed a significant reduction in starch levels in *oscin1/2*, with slight reductions in the *oscin1* and *oscin2* single mutants (Figure 3D and Figure 6A). The complement line did not accumulate starch to the same extent as the *oscin2* mutant, which aligns with our staining observations (Figure 3G). We also found a significant reduction in sucrose content in the *oscin1/2* mutant (Figure 6A), suggesting that the sucrose supply to the pollen is impaired, likely contributing to the low-starch phenotype.

Because *CIN* is expressed in the apoplastic region, we measured soluble sugars in the locular fluid, which is considered the apoplastic space of the anthers. The sucrose levels remained constant across the WT, *oscin1*, *oscin2*, and *oscin1/2*, while the glucose and fructose levels were found to be decreased. The glucose and fructose levels in *oscin1/2* were significantly lower than those in the single mutants (Figure 6B). Sucrose is continuously supplied to the anthers via the panicle branch, maintaining a steady concentration in both the WT and all the mutants examined. However, the decreased CIN activity, which is necessary to degrade sucrose, in the mutants likely explains the observed reduction in hexose levels.

In seeds, the sucrose levels were decreased three days after fertilization in *oscin2*, *oscin1/2*, and Comp #2. The glucose and fructose levels decreased only in *oscin1/2* seeds (Figure 6C). The starch levels were significantly reduced in *oscin2*, *oscin1/2*, and Comp #2, with a slight reduction observed in *oscin1* (Figure 6D). These findings are consistent with the observed seed phenotypes and measured CIN activity levels in seeds (Figure 4 and Figure 5B). Studying seeds is crucial because proper sucrose metabolism and allocation during early seed development are essential for successful grain filling and crop yields. The significant reductions in starch and sugar content observed in the seeds of the mutants underscore the vital role of CIN in carbohydrate metabolism during early seed formation.

## 3. Discussion

Sucrose is transported from sources (mature leaves) to sinks (importing tissues such as roots, stems, anthers, and seeds) through the phloem tissues in veins [17]. In sink tissues, sucrose must pass through the apoplast to reach filial tissues. CIN, the only enzyme known to break down sucrose in the apoplast, co-localizes with sucrose in this space and hydrolyzes it into hexoses.

In rice, there are nine CIN isoforms, each exhibiting highly distinct expression patterns across different tissues [18]. This tissue-specific expression suggests that each isoform is likely regulated differently, depending on the tissue in which it is expressed. Given the critical nature of carbon partitioning, it is plausible that different combinations of CIN isoforms are responsible for distributing sucrose to various tissues. Despite the potential significance of these isoforms, the functions of individual CIN forms have been scarcely studied. In this research, we identified two CIN isoforms specifically expressed in the anther, *OsCIN1* and *OsCIN2*, and aimed to investigate their functions. By focusing on their roles in pollen development and seed formation, we highlight the importance of CIN activity in sucrose metabolism within the reproductive tissues of rice.

### 3.1. Maternal-Genotype-Dependent Seed Phenotype in oscin1/2

Because *OsCIN1* and *OsCIN2* show very low expression in pollen grains at the mature stage but are highly expressed in the surrounding anther tissues, we hypothesized that these genes function in the apoplast, facilitating the movement of sucrose from maternal tissues to filial tissues. Therefore, we conducted a functional analysis of these genes. *OsCIN2* is also known as *GIF1*, and its mutation is known to cause improper grain filling. Previous studies have shown that *gif1* produces lighter seeds than the WT and exhibits an opaque seed phenotype. *GIF1* is expressed in the ovular vascular and lateral stylar vascular traces, both of which are part of the maternal tissue. We isolated a T-DNA insertion loss-of-function mutant for *OsCIN1*, but it did not exhibit any visible phenotypes. Given the specific expression of both *OsCIN1* and *OsCIN2* in the anthers, we anticipated additional phenotypes beyond those observed in *gif1* and thus generated the *oscin1/2* double mutant through genetic crossing. The *oscin1/2* plants exhibited low fertility and produced shrunken seeds. However, due to the very low fertility of all the selected *oscin1/2* plants from the F2 segregants, we were unable to obtain a sufficient number of seeds for further experiments. In an attempt to secure more *oscin1/2* seeds, we focused on selecting shrunken seeds, which we expected to account for 25% of the seeds from the *oscin1/OsCIN2^+/–^* plants. Surprisingly, we found very few shrunken seeds, and many of those shrunken seeds did not carry the *oscin1/2* genotype. This observation led us to identify a possible discrepancy between seed genotype and phenotype. Further genetic crosses, particularly using the WT (*OsCIN1^+/+^/OsCIN2^+/+^*) as the pollen donor and *oscin1/2* as the maternal parent, resulted in all the seeds exhibiting a shrunken phenotype, despite their genotype being *OsCIN1^+/−^*/*OsCIN2^+/−^* (Table 1). These results clearly indicate that the shrunken-seed phenotype is determined by the genotype of the maternal tissue rather than the filial tissue. Given that seeds rely on external sources of nutrients, it is highly likely that the flow of sugars from the maternal tissues directly influences the carbohydrate levels in developing seeds.

### 3.2. Sugar Transport Pathway in Apoplast Between Maternal and Filial Tissue

Recent studies have shown that a double mutant for *OsSWEET11a* and *OsSWEET11b* results in male sterility and a shrunken-seed phenotype. The expression of *OsSWEET11a* and *OsSWEET11b* in the anther veins strongly supports their roles as key sugar transporters responsible for unloading sucrose into the apoplast between maternal and filial tissues in rice [6]. Sucrose unloaded from the maternal tissue into the apoplast must be delivered to the filial tissue, but it remains unclear whether this occurs via sucrose itself, hexose, or both. Currently, there is no definitive genetic evidence identifying the sugar transporters involved in this process. In this study, we confirmed that some of the sucrose unloaded into the apoplast must be hydrolyzed into hexose to properly supply carbohydrates to filial tissues, such as pollen grains and seeds. However, the severe starch accumulation defects in pollen grains and the more pronounced shrunken-seed phenotype of the *ossweet11a/ossweet11b* mutants [6], compared to those of *oscin1/2*, suggest that not all sucrose is broken down into hexose before reaching the filial tissue. This implies the presence of both sucrose and monosaccharide transporters in the pollen grains and seed coats. Functional analysis of candidate sucrose and monosaccharide transporters that are highly expressed in pollen grains [16] may help further resolve how sugars are allocated between maternal and filial tissues.

The CIN activity in the anthers and seeds of the WT was over 500 times and 5 times higher, respectively, than that in the leaves (Figure 5). For sucrose to move from mesophyll cells to sink organs, a concentration gradient is required, necessitating a reduction in sucrose concentration in the destination tissues. CIN likely plays a crucial role in lowering sucrose concentrations in the apoplast between source and sink tissues, enabling the uniporter SWEET transporters to efficiently unload sucrose into the apoplast. In source organs, the CIN activity is kept low to prevent sucrose degradation, allowing efficient transport to sink organs. In the sink tissues, CIN activity is likely regulated based on the sugar demand of each tissue, facilitating sucrose unloading. The overexpression of *GIF1* resulted in a shrunken-seed phenotype, further supporting the importance of the precise localization and regulation of CIN for normal seed development [15]. Although the CIN activity in the anthers of Comp #2 was comparable to that of *oscin1*, pollen starch did not accumulate to the same extent as in *oscin1* (Figure 5A and Figure 6A), which can be explained by this regulatory mechanism. Thus, CIN plays a crucial role not only in hydrolyzing sucrose into hexose for supplying carbohydrates to sink tissues but also in creating a low sucrose concentration in the apoplast, which is essential for the continuous unloading of sucrose via SWEETs.

### 3.3. Fine-Tuning of CIN Activity in Various Tissues

The regulation of CIN activity is thought to occur not only at the transcriptional level but also through post-translational modifications. Based on the expression patterns of the nine *OsCIN* genes, their expression levels in leaves do not significantly differ from those in seeds or anthers (Figure S1). Given that the combined expression levels of all nine *OsCIN* genes are quite similar across tissues, it seems unlikely that the significantly higher CIN activity observed in anthers and seeds is solely due to differences in gene expression. While this phenomenon has not been reported in rice, CIN inhibitors have been identified in other species such as *Arabidopsis thaliana* [19], *Glycine max* [20], *Ipomoea batatas* [21], *Saccharum officinarum* [22], *Solanum lycopersicum* [23], and *Zea mays* [24]. Given that the sugar supply to sink organs fluctuates based on environmental factors, it is plausible that CIN activity is regulated not only through transcriptional control but also post-translationally, including via modulation by competitive cell inhibitors that bind to the active site of the invertase enzyme [25]. The distribution of sucrose is likely determined by the tissue-specific expression and levels of various *CIN* isoforms, in combination with the presence and regulation of CIN inhibitors in each tissue. Understanding this regulatory mechanism could provide valuable insights for manipulating carbohydrate allocation to sink organs, potentially enhancing crop yields. Additionally, this knowledge may help mitigate fertility reductions caused by decreased CIN activity under stressful conditions such as high temperature or drought.

## 4. Materials and Methods

### 4.1. Isolation and Growth of Rice Mutants

The *oscin1* mutant, carrying a T-DNA insertion in the *OsCIN1* gene, was identified from the segregants of a T-DNA-tagged mutant line, 3A-02853, in the cv. Dongjin background [26]. The genotypes of the mutant plants were determined through the PCR analysis of genomic DNA using *OsCIN1* gene-specific primers, CIN1F and CIN1R, and a T-DNA-specific RB primer (Supplementary Table S1). The genotype of the *oscin2* mutant, containing a single-nucleotide deletion causing a frameshift, was obtained from Professor Zuhua He [15] and confirmed via Sanger sequencing using amplicons generated with CIN2F and CIN2R primers (Supplementary Table S1). Plants were grown either in a greenhouse with a light/dark cycle (30/20 °C) of 14 h/10 h or in an experimental field plot under natural environmental conditions during the summer.

### 4.2. Genetic Complementation Experiment

Full-length *OsCIN1* cDNA from cv. Dongjin wild type (WT) was amplified via PCR using CIN1 Full-F and CIN1 Full-R primers (Supplementary Table S1). The full-length cDNA of *OsCIN1* was subsequently cloned into the binary vector pPZPHa3(+). The resulting construct was introduced into Dongjin WT rice calli via *Agrobacterium*-mediated transformation. Homozygous transgenic lines were crossed with *oscin1/2* mutants, and *oscin1/oscin2/p35S:OsCIN1* plants were selected through genomic DNA PCR.

### 4.3. RNA Isolation and RT-PCR Analysis

Anthers at various developmental stages were categorized based on their length and appearance, following the criteria established by Yi et al. [27]. Total RNA was extracted from the collected anthers using Trizol reagent (Invitrogen, Gaithersburg, MD, USA) and reverse-transcribed into cDNA using the ReverTra Ace qPCR RT Master Mix cDNA Synthesis Kit (Toyobo, Tokyo, Japan). Quantitative real-time RT-PCR (RT-qPCR) was conducted using Prime Q-Master Mix with SYBR Green I (Thermo Fisher Scientific, Waltham, MA, USA) on a Rotor-Gene 6000 real-time amplification system (Corbett Research, Sydney, Australia). cDNA synthesized from the total RNA of anthers at different developmental stages served as templates. The data were normalized using the *OsUBQ5* gene (LOC_Os01g22490) as an internal control. The sequences of the primers used for RT-qPCR are listed in Supplementary Table S1.

### 4.4. Histochemical Thin Section Analysis

Anthers at various developmental stages were prepared for sectioning according to the protocol described by Moon et al. [28]. Briefly, harvested anthers were fixed in a formalin–acetic acid–alcohol solution for 8 h, embedded in Technovit^®^ 8100 resin (Kulzer, Hanau, Germany), and sectioned to a thickness of approximately 10 μm using a rotary microtome (Leica RM2165, Leica Microsystems, Wetzlar, Germany). The sections were stained with 0.1% toluidine blue O (Sigma-Aldrich, St. Louis, MO, USA) dissolved in 1% sodium chloride solution and observed under an Olympus BX61 microscope (Olympus, Tokyo, Japan).

### 4.5. Pollen Staining and Light Microscopy

Pollen nuclei were stained by incubating pollen grains in phosphate-buffered saline containing 0.5 μg/mL Hoechst 33342 (Sigma) for 1 h at 65 °C. The pollen starch was stained using 10% (*v*/*v*) Lugol’s solution (Sigma). The nucleus- and starch-stained pollen grains were observed under UV light and white light, respectively, using an Olympus BX61 microscope.

### 4.6. Measurement of Cell Wall Invertase Activity

CIN activity was assayed according to the method described by Ruhlmann et al. [29]. Fresh samples from various tissues were collected and flash-frozen in liquid nitrogen. The samples were transferred to 1.5 mL microcentrifuge tubes containing 400 μL of extraction buffer (50 mM HEPES-NaOH, pH 8.0; 5 mM MgCl_2_; 2 mM EDTA; 1 mM MnCl_2_; 1 mM CaCl_2_; 1 mM dithiothreitol; and 0.1 mM phenylmethylsulfonyl fluoride) and homogenized with a pestle, followed by incubation on ice for 10 min. The samples were then centrifuged at 13,000× *g* for 10 min at 4 °C. The pellets were washed three times with 400 μL of extraction buffer via resuspension and centrifugation at 13,000× *g* for 10 min at 4 °C. A final wash was performed using 400 μL of 80 mM sodium acetate buffer (pH 4.8), after which the pellets were resuspended in 400 μL of 80 mM sodium acetate buffer (pH 4.8). The CIN activity was measured by incubating 25 μL of the extract in a reaction mixture (250 μL) containing 80 mM sodium acetate buffer (pH 4.8) and 25 mM sucrose. After a 30 min incubation, the reaction was stopped by immersing the tubes in a boiling water bath for 4 min. The glucose content of 100 μL of the reaction mixture was then determined enzymatically.

### 4.7. Determination of Soluble Sugars and Starch

Fresh samples from various tissues were harvested and extracted using 10% perchloric acid. The insoluble and soluble fractions were separated by centrifugation at 20,000× *g* for 5 min. The soluble fraction was neutralized to a pH of 6–7 with 2 M KOH containing 0.4 M MES buffer. The glucose, fructose, and sucrose concentrations were determined through an NAD(P)H-coupled enzymatic assay by sequentially adding 0.5 units of hexokinase, 2 units of isomerase, and 10 units of invertase. The measured metabolite contents were then normalized to the fresh weights of the samples. The insoluble fraction was washed several times with 80% ethanol until the green coloration of the plant tissue was completely removed. The pellet was resuspended in water, and the starch was gelatinized at 95 °C for 15 min. The solution was adjusted to a final concentration of 100 mM sodium acetate (pH 4.8), 1 unit of amyloglucosidase, and 1 unit of α-amylase, followed by incubation at 37 °C for 2 h. After incubation, the mixture was centrifuged at 20,000× *g* for 5 min, and the glucose content in the supernatant was quantified using a NAD(P)H-coupled enzymatic assay to determine the starch content [30].

## 5. Conclusions

Our findings highlight the critical role of CIN in regulating sucrose metabolism and its allocation between maternal and filial tissues during reproductive development in rice. The distinct expression patterns and regulatory mechanisms of *OsCIN1* and *OsCIN2* in reproductive tissues suggest that these isoforms play a crucial role in pollen development and seed formation. The male-sterility and shrunken-seed phenotypes observed in the *oscin1/2* mutants, factors determined by the maternal genotype, underscore the importance of sucrose transport and hydrolysis in maintaining proper carbohydrate flow to developing pollen and seeds. Further investigation of CIN isoforms and their regulatory mechanisms, including post-translational modifications and the role of potential CIN inhibitors, will be essential for optimizing carbohydrate partitioning to enhance crop fertility and yields, especially under environmental stress conditions.

## Figures and Tables

**Table 1:** Genetic segregation and seed phenotypes in the progeny of F1 plants from various mutant crosses. Genotypes of progeny plants were determined through genomic DNA PCR using T-DNA- and gene-specific primers for *oscin1* and through DNA sequencing for *oscin2*. *OsCIN1^+/+^* or *OsCIN2^+/+^*, wild-type genotype for the respective genes; *OsCIN1^+/−^* or *OsCIN2^+/−^*, heterozygous genotype for the respective genes; *p35S:OsCIN1^+/+^*, homozygous insertion of *p35S:OsCIN1*; *p35S:OsCIN1^+/−^*, heterozygous insertion of *p35S:OsCIN1*; *p35S:OsCIN1^−/−^*, no insertion of *p35S:OsCIN1*.

Parents Plants		Genotype of Progeny Observed/Expected Genotype of Progeny in % (Number of Observed/Number of Analyzed Seedlings//Observed/Expected Seed Phenotype)		
Paternal Parent	Maternal Parent			
*oscin1/OsCIN2^+/–^*	*oscin1/OsCIN2^+/–^*	*oscin1/OsCIN2^+/+^* 25.5%/25% (53/208//normal/normal)	*oscin1/OsCIN2^+/–^* 51.4%/50% (107/208//normal/normal)	*oscin1/oscin2* 23.1%/25% (48/208//normal/shrunken)
*OsCIN1^+/–^/oscin2*	*OsCIN1^+/–^/oscin2*	*OsCIN1^+/+^/oscin2 * 24.7%/25% (46/186//opaque/opaque)	*OsCIN1^+/–^/oscin2 * 52.2%/50% (97/186//opaque/opaque)	*oscin1/oscin2* 23.1%/25% (43/186//opaque/shrunken)
*oscin1/oscin2/p35S:OsCIN1^+/–^*	*oscin1/oscin2/p35S:OsCIN1^+/–^*	*oscin1/oscin2/p35S:OsCIN1^+/+^* or *oscin1/oscin2/p35S:OsCIN1^+/–^* 76%/75% (152/200//opaque/opaque)		*oscin1//oscin2//p35S:OsCIN1-OX^–/–^* 24%/25% (48/200//opaque/shrunken)
*OsCIN1^+/+^/OsCIN2^+/–^*	*oscin1/oscin2*	*OsCIN1^+/–^/OsCIN2^+/–^* 100%/100% (198/198//shrunken/normal)		

## Data Availability

No new data were created or analyzed in this study. Data sharing is not applicable to this article.

## Supplementary Materials

The following supporting information can be downloaded at https://www.mdpi.com/article/10.3390/ijms252111557/s1. Reference [31] is cited in the Supplementary Materials.
