# Supplementary material for: Cell Wall Invertases from Maternal Tissues Modulate Sucrose Flux in Apoplastic Pathways During Rice Anther and Seed Development
Source: Int J Mol Sci. 2024 Oct 28;25(21):11557. doi: 10.3390/ijms252111557 (PMC11546591; doi:10.3390/ijms252111557)
Supplement: Supplementary file 1 [file ijms-25-11557-s001.zip › ijms-3265829-supplementary.pdf]

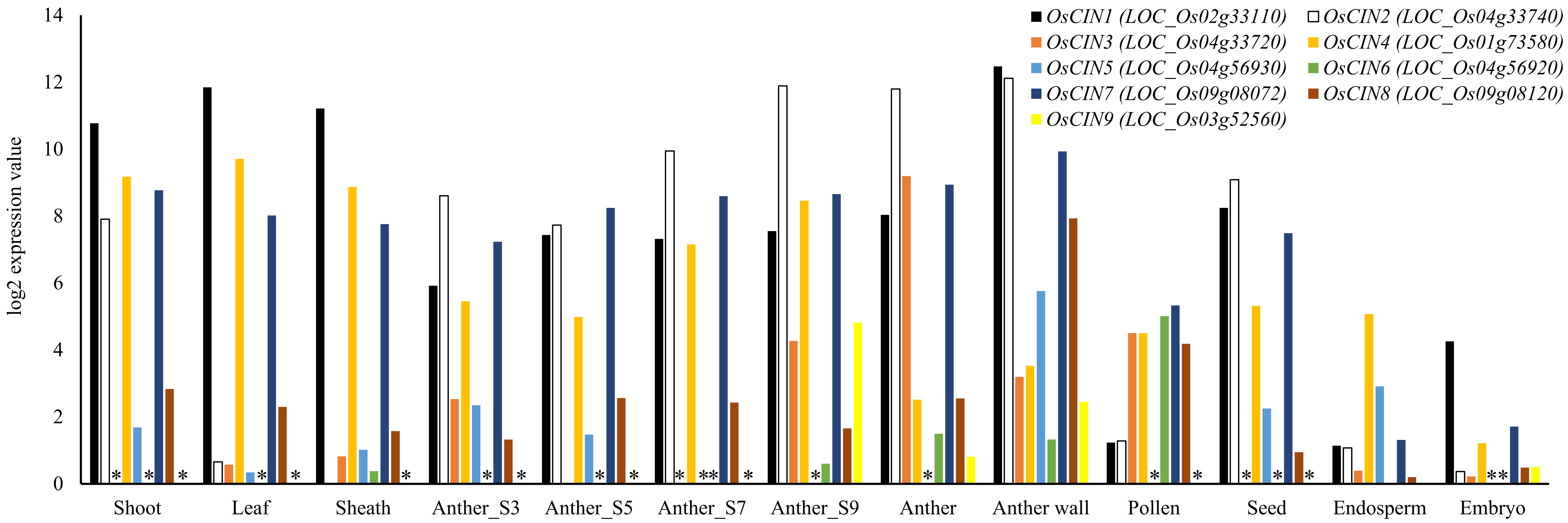

Figure S1. Digital expression profile of *OsCIN* genes. The information was extracted from CAFRI-Rice (https://cafri-rice.khu.ac.kr/; Hong *et al*., 2020. CAFRI-Rice: CRISPR applicable functional redundancy inspector to accelerate functional genomics in rice. The Plant Journal). * = not detectable

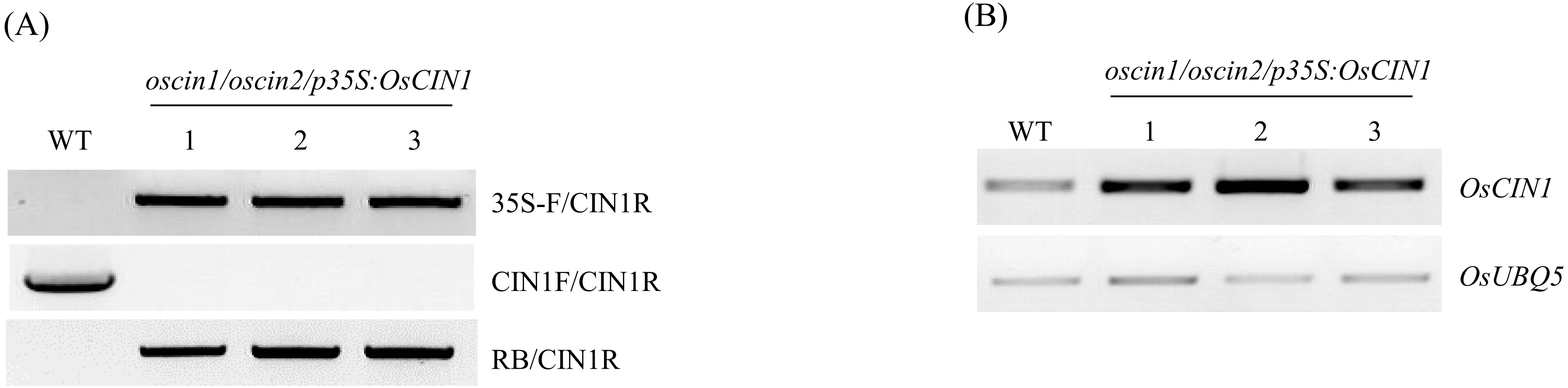

Figure S2. Genomic DNA PCR (A) and RT-PCR (B) analysis of *oscin1/oscin2/p35S:OsCIN1* plants, the genetically complemented lines. 35S-F/CIN1R primer set was used for detecting *OsCIN1-OX* transgene*,* CIN1RF/CIN1R set for WT genomic copy, and LB/CIN1R set for T-DNA insertion. *OsUBQ5* was used as an endogenous control. WT, wild type

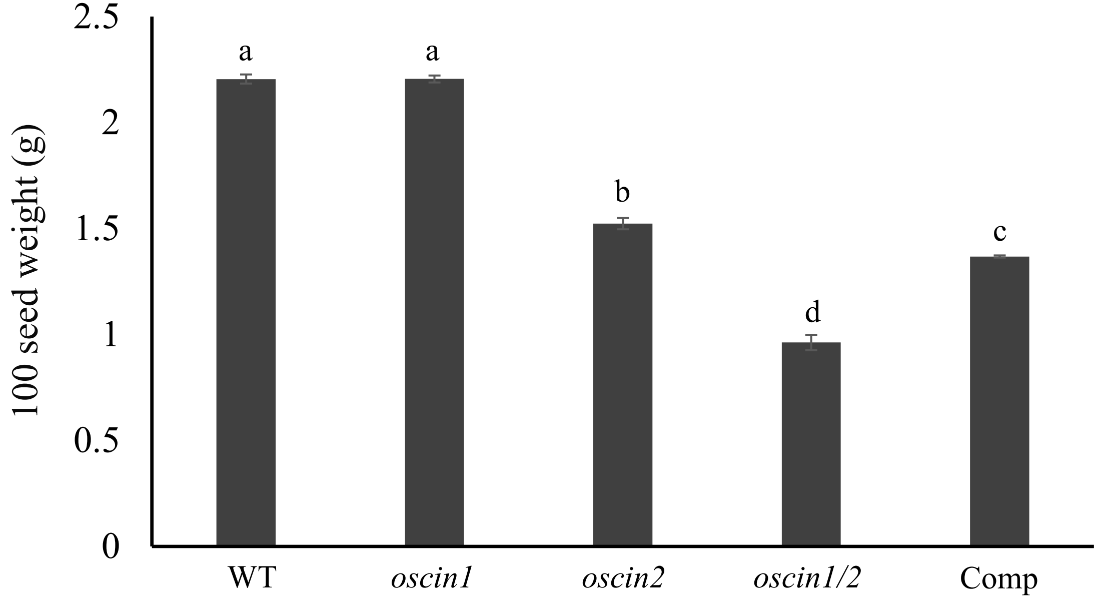

Figure S3. Seed weight of *OsCIN* mutants. All the data represent means (± SEs) from at least three different plants. Significant differences between groups were determined using Tukey's HSD test for multiple comparisons. Different uppercase letters indicate significant differences ($p < 0.05$).
